# Too much of a good thing

**DOI:** 10.7554/eLife.41178

**Published:** 2018-09-17

**Authors:** H Lee Sweeney

**Affiliations:** 1 Myology InstituteUniversity of FloridaGainesvilleUnited States; 2 Department of Pharmacology and TherapeuticsUniversity of FloridaGainesvilleUnited States

**Keywords:** muscle fiber, myosin, cardiomyopathy, muscle contraction, *D. melanogaster*

## Abstract

A mutation that causes heart disease in humans increases the number of active myosin heads during muscle contraction in fruit flies, leading to the progressive dysfunction of the flight muscles and heart tube.

**Related research article** Kronert WA, Bell KM, Viswanathan MC, Melkani GC, Trujillo AS, Huang A, Melkani A, Cammarato A, Swank DM, Bernstein SI. 2018. Prolonged cross-bridge binding triggers muscle dysfunction in a fly model of myosin-based hypertrophic cardiomyopathy. *eLife* **7**:e38064. doi: 10.7554/eLife.38064

Our muscles help us to move, eat, breathe and pump blood through our body. The skeletal and heart muscles of vertebrates consist of bundles of muscle fibers, with each fiber containing thousands of smaller structures called myofibrils. Within these myofibrils, thick myosin filaments and thin actin filaments can slide past each other to change the length of the muscle. This involves a part of a myosin filament, called the myosin head, 'grabbing hold' of an actin filament.

Controlling the interactions between the myosin heads and actin is a finely tuned and highly regulated process, and even small changes to it can lead to dysfunction and disease. For example, in a condition called hypertrophic cardiomyopathy, mutations in the myosin head can cause the walls of the heart to thicken, ultimately restricting the outflow of blood (Garfinkel et al., 2018). So far, it has been unclear how the mutations can cause these problems. Now, in eLife, Sanford Bernstein, Douglas Swank and colleagues at San Diego State University, Rensselaer Polytechnic Institute and Johns Hopkins University – including William Kronert as first author – report the surprising finding that a mutation in the motor portion of the myosin head that causes human hypertrophic cardiomyopathy can lead to degeneration of the flight muscles as well as the dysfunction of the heart tube in fruit flies (Kronert et al., 2018).

What appears to underlie this seemingly perplexing result is a loss of proper interactions either within myosin heads or, possibly, between the myosin heads and other components of the myosin filament. These interactions normally lead to the myosin heads being packed onto the ‘backbone’ of the myosin filament in a way that restricts their interactions with the actin filaments.

The myosin head contains a motor domain, a regulatory light chain and an essential light chain. The key structural insights into the packing of the myosin heads came from studying the regulation of smooth muscle myosin in vertebrates, which is inactive unless the regulatory light chain is phosphorylated (Wendt et al., 2001). This work revealed asymmetric interactions (known as the 'interacting head motif’) between neighboring myosin heads, which trapped both heads in inactive conformations: the actin binding site of one head was blocked by binding to the other in a way that prevented the latter from releasing the products of ATP hydrolysis when it interacted with actin (Figure 1). Interacting head motifs have also been found in the skeletal muscle of tarantulas, the heart muscles of vertebrates, and even in primitive animals that do not form muscles (Alamo et al., 2016; Woodhead et al., 2005; Lee et al., 2018; Zoghbi et al., 2008).

**Figure 1.**
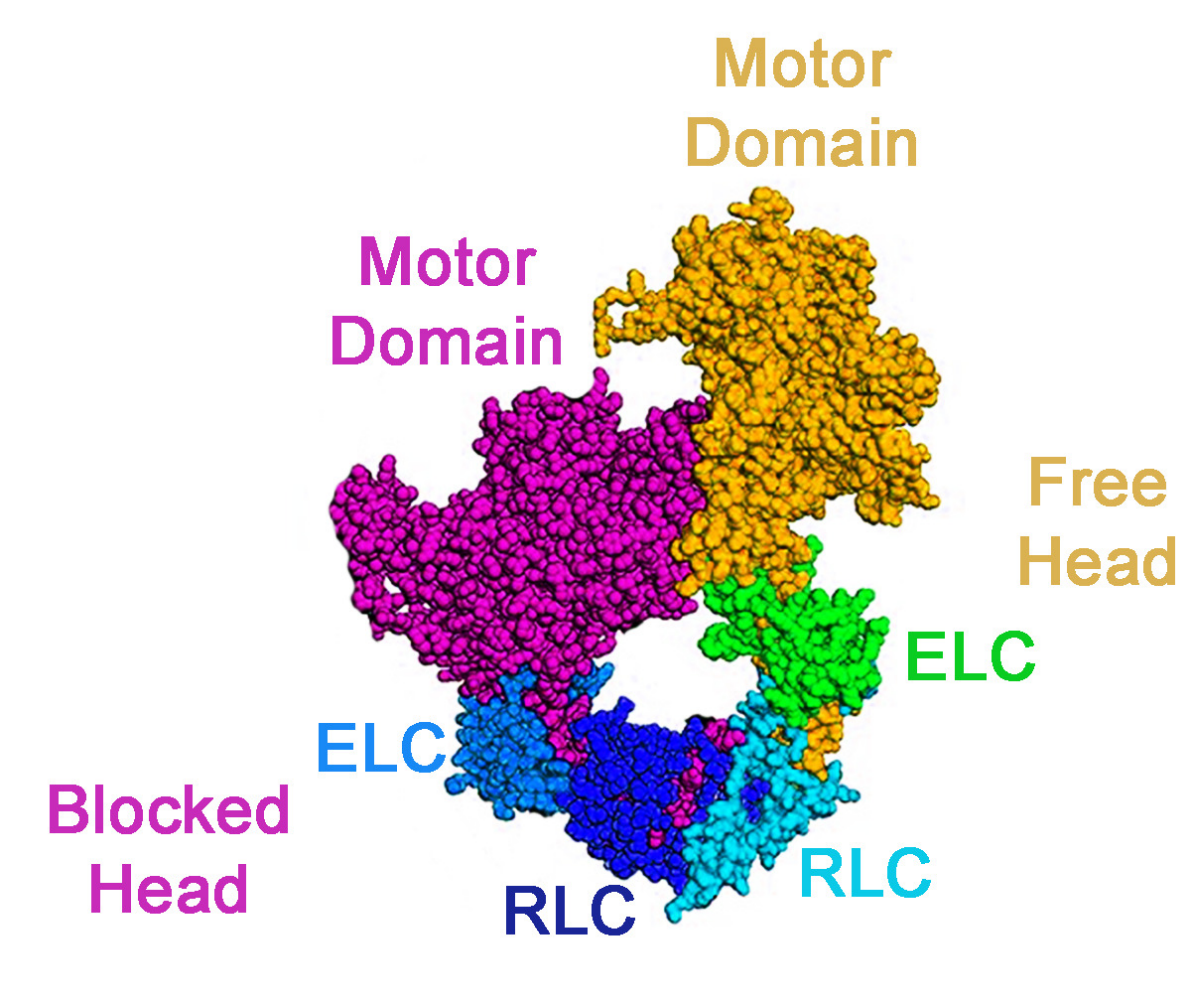
An interacting head motif. A schematic view of an interacting head motif: the motor domain of one myosin head (the blocked head, shown in purple) has its actin binding site blocked by interactions with the second head (the free head). The essential light chain (ELC) and regulatory light chain (RLC) of each myosin head are also involved in the formation of the interacting head motif.

In an energy conserving ‘super-relaxed state’, myosin is trapped in a state with very low basal ATPase activity (Stewart et al., 2010). This appears to be a mechanism to stabilize myosin in its ‘pre-powerstroke’ state, in which inorganic phosphate and ADP are confined within the myosin head. Kronert et al. suggest that this pre-powerstroke state is destabilized by the mutation they were studying.

The researchers used fruit flies with a specific mutation on the side of the myosin head, called K146N, which causes thickening of the heart wall in humans. In the flies, the mutation leads to a degenerated heart tube and flight muscles. For both humans and flies, the problem appears to be the same: too many myosin heads interact during contraction and attach to actin for longer than usual, leading to hyperactivation, damage and disorder in the muscle. Myosin was able to create more force, while the movement of actin slowed down. Over time, the increased cost of performing work caused the muscles to degenerate. Although this does not lead to hypertrophy of the flight muscles – as it does in the human heart – there is decreased relaxation and restriction of the heart tube in the flies, paralleling human hypertrophic cardiomyopathy.

The ‘myosin mesa’ theory hypothesizes that many of the mutations causing hypertrophic cardiomyopathy are surface mutations that interfere with the formation and/or stabilization of the interacting head motif (Trivedi et al., 2018). It also proposes that a subset of these mutations might lead to interactions with another protein of the thick filament of the heart muscle, the myosin-binding protein C, which could help stabilize the interactions between the two myosin heads in the interacting head motif. This in turn could explain why mutations in myosin-binding protein C can also cause hypertrophic cardiomyopathy. Based on these concepts, a drug that stabilizes the super-relaxed state and the interacting head motif is being developed for the treatment of human hypertrophic cardiomyopathy (Anderson et al., 2018).

All in all, fruit flies may be a surprisingly good model to test the effects of mutations associated with human hypertrophic cardiomyopathy, apart from those that may interact directly with myosin-binding protein C, which is not present in fruit flies. However, the major significance of the work is that it underscores that since the super-relaxed state and the packing of interacting head motifs onto thick filaments is conserved throughout the evolution of muscle, it is likely that their functions – which would be to control the number of myosin heads available to interact with actin when the muscle is activated, and to lower the energetic cost when the muscle is relaxed – are also conserved.
